# The contribution of attention-deficit/hyperactivity disorder polygenic load to metabolic and cardiovascular health outcomes: a large-scale population and sibling study

**DOI:** 10.1038/s41398-024-03178-2

**Published:** 2024-11-13

**Authors:** Ebba Du Rietz, Tian Xie, Rujia Wang, Rosa Cheesman, Miguel Garcia-Argibay, Zihan Dong, Jia Zhang, Jacobien Niebuur, Melissa Vos, Harold Snieder, Henrik Larsson, Catharina A. Hartman

**Affiliations:** 1 Department of Medical Epidemiology and Biostatistics, Karolinska Institute, Stockholm, Sweden; 2 Interdisciplinary Center Psychopathology and Emotion Regulation (ICPE), Department of Psychiatry, University Medical Center Groningen, University of Groningen, Groningen, The Netherlands; 3 Department of Epidemiology, University of Groningen, University Medical Center Groningen, Groningen, The Netherlands; 4 PROMENTA Research Center, Department of Psychology, University of Oslo, Oslo, Norway; 5 School of Medical Sciences, Faculty of Medicine and Health, Örebro University, Örebro, Sweden; 6 Shenzhen Center for Chronic Disease Control, Shenzhen, Guangdong China

**Keywords:** ADHD, Genomics

## Abstract

Emerging evidence suggests that ADHD is associated with increased risk for metabolic and cardiovascular (cardiometabolic) diseases. However, an understanding of the mechanisms underlying these associations is still limited. In this study we estimated the associations of polygenic scores (PGS) for ADHD with several cardiometabolic diseases and biomarkers. Furthermore, we investigated to what extent the PGS effect was influenced by direct and indirect genetic effects (i.e., shared familial effects). We derived ADHD-PGS in 50,768 individuals aged 18–90 years from the Dutch Lifelines Cohort study. Using generalised estimating equations, we estimated the association of PGS with cardiometabolic diseases, derived from self-report and several biomarkers measured during a physical examination. We additionally ran within-sibling PGS analyses, using fixed effects models, to disentangle direct effects of individuals’ own ADHD genetic risk from confounding due to indirect genetic effects of relatives, as well as population stratification. We found that higher ADHD-PGS were statistically significantly associated with several cardiometabolic diseases (R-squared [R^2^] range = 0.03–0.50%) and biomarkers (related to inflammation, blood pressure, lipid metabolism, amongst others) (R^2^ range = 0.01–0.16%) (*P* < 0.05). Adjustment for shared familial factors attenuated the associations between ADHD-PGS and cardiometabolic outcomes (on average 56% effect size reduction), and significant associations only remained for metabolic disease. Overall our findings suggest that increased genetic liability for ADHD confers a small but significant risk increase for cardiometabolic health outcomes in adulthood. These associations were observable in the general population, even in individuals without ADHD diagnosis, and were partly explained by familial factors shared among siblings.

## Introduction

ADHD is a common and highly heritable neurodevelopmental disorder with an estimated worldwide prevalence of 3–7% in childhood and adolescence [1, 2], and around 3% in adulthood [3, 4]. While there is a plethora of research documenting the frequent co-occurrence of psychiatric disorders with ADHD [5], associations with physical disorders, especially those that occur in older age, have been less studied. This is partly due to the lack of available data on ageing adults with ADHD. Recent evidence from large-scale studies have reported associations between ADHD and increased risk of metabolic and cardiovascular (CVD) diseases (cardiometabolic diseases) [6–9], which are some of the major causes of disability globally and leading causes of death [10, 11]. However, these studies have often included a limited number of cardiometabolic outcomes and have mainly captured adults who have received a clinical diagnosis of ADHD. This is a potential problem due to the low prevalence of ADHD diagnoses in older adults, most likely due to underdiagnosis [3]. Studies using genetic data may be useful to overcome this problem, as polygenic scores can act as proxy for continuous ADHD risk in the general population instead of relying on diagnoses.

A limited number of genetic studies, using family-based and molecular genetic designs, have suggested genetic correlations between ADHD and a number of cardiometabolic outcomes [6, 12, 13]. A recent genetic study revealed weak-to-moderate genetic correlations of ADHD with BMI, coronary artery disease and levels of triglycerides and HDL cholesterol [12]. However, conclusive and well-powered studies are still lacking. The reported genetic correlations may reflect shared biology, whereby the same genetic variants that increase the risk for ADHD also directly increase risk for cardiometabolic conditions, or causality, where ADHD may have causal effects on cardiometabolic conditions. Furthermore, it is largely unknown to what extent estimates of the effect of genetic risk of ADHD on cardiometabolic health are confounded by familial genetic effects, by which parental genetic risk for ADHD correlates with family-environmental factors (so called ‘genetic nurture’) that in turn increase risk for cardiometabolic outcomes in offspring (e.g., socioeconomic status [SES], lifestyle, diet, neighbourhood, household chaos). Within-sibling PGS models take advantage of the random segregation of genetic material at meiosis. Indeed, within-sibship differences in PGS must be due to random genetic inheritance and are not correlated with confounders shared by siblings. Therefore, sibling models are thought to separate out direct genetic effects (i.e. causal individual genetic effects originating from individual genome) from indirect genetic effects (familial genetic effects originating in the genome of family member, independent of genetic transmission), population stratification (systematic allele frequency differences according to ancestry) and assortative mating.

The aim of this large-scale population-based study was to estimate the genetic associations between ADHD and cardiometabolic diseases as well as a range of related biomarkers (e.g. cholesterol, glucose levels, blood pressure). Insight into the links between ADHD and different cardiometabolic biomarkers may provide a better understanding of the biological mechanisms that are implicated in individuals with adult ADHD. It is important for research, in general, to establish these underlying mechanisms, as the knowledge could in the long run guide clinical practice and prevention strategies relating to cardiovascular health issues in individuals with ADHD. We further aim to control for shared familial factors, using a within-sibling design, and investigate moderating factors (age, sex and SES) of the genetic association between ADHD and cardiometabolic outcomes.

## Materials and methods

### Sample and data collection

Lifelines is a multi-disciplinary prospective population-based cohort study examining in a unique three-generation design the health and health-related behaviours of 167,729 persons living in the North of the Netherlands. It employs a broad range of investigative procedures in assessing the biomedical, socio-demographic, behavioural, physical and psychological factors which contribute to the health and disease of the general population, with a special focus on multi-morbidity and complex genetics [14]. Between 2006 and 2013, randomly selected general practitioners invited all their listed patients aged 25-50 years to participate in the study. Self-reported questionnaires collected information on demographics, family composition, work and education, health and pharmacological treatment (using Anatomical Therapeutic Chemical Classification System [ATC]). Blood and urine samples, blood pressure and BMI were obtained during a physical examination. In the current study, we used only baseline data and the following exclusion criteria: age <18, missing genetic data or non-European ancestry. The final sample of *N* = 50,768 participants was included in the analyses (see Supplementary Fig. 1 for flow chart of genetic data processing). The authors assert that all procedures contributing to this work comply with the ethical standards of the relevant national and institutional committees on human experimentation (approved by the Medical Ethical Committee at the University Medical Center Groningen) and with the Helsinki Declaration of 1975, as revised in 2008. Informed consent was obtained from all participating individuals.

**Fig. 1:**
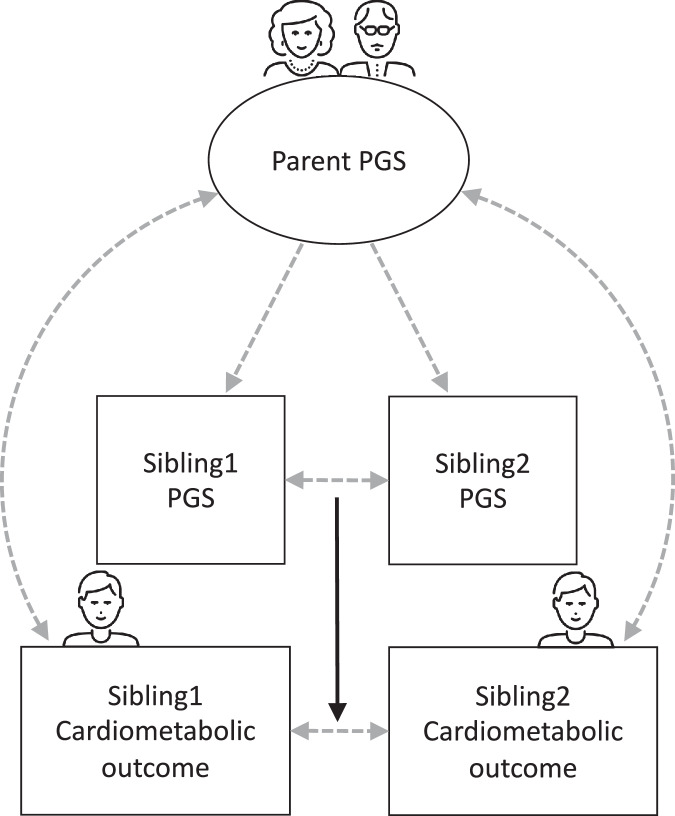
Analytical sibling design to estimate direct genetic effects. Note: Square = observed variable, circle = unobserved/latent variable; direct genetic effects (controlling for indirect genetic effects) are represented with solid arrows. Icons from. www.thenounproject.com.

### Cardiometabolic disease

Using questionnaires, participants were asked to report the presence of cardiometabolic diseases. The definition of diseases was based on the structure of the International Classification of Diseases version 10 (ICD-10) [15]. In line with past publications from Lifelines, operationalization methods were developed to define four cardiometabolic diseases: myocardial infarction, heart failure, atrial fibrillation [16] and type-2 diabetes [17]. These involved self-reported disease validated with biomarkers or cardiovascular/diabetic medication (self-report) (Supplementary Table 1 for definitions). Obesity was defined as having a BMI of 30 or above, measured using height and weight (kg/m^2^) obtained at the physical examination.

### Cardiometabolic biomarkers

At the baseline assessment, participants were invited to visit one of twelve Lifelines Research sites to undergo a physical examination and a series of tests. Blood pressure was measured ten times over a 10-min period, and the registered blood pressure was the average of the final three readings in millimetres of mercury (mmHg). At the research sites, blood and 24-hour urine were collected from participants and transported to the central Lifelines laboratory in Groningen [16]. We included 27 biomarkers (obtained during the physical examination), related to cardiometabolic health across different functional groups: glucose metabolic, red blood cells, lipid metabolism, liver function, kidney function, thyroid, inflammation and blood pressure.

Self-reported medication at baseline was used to extract information on individuals who were on medication for cholesterol- or blood pressure-lowering medication and diabetic medication (insulin or/and tablets). Following standard measures, individuals on cholesterol-lowering medication had their pre-medication levels approximated by dividing the LDL cholesterol value by 0.7 and the triglycerides value by 0.8 [18]. We adjusted blood pressure values for medication use by adding 15 and 10 mmHg to systolic and diastolic blood pressure, respectively, for individuals reported to be taking blood pressure-lowering medication [19]. We excluded the glucose and haemoglobin A1c (HbA1c) measures for individuals on diabetic medication.

In order to avoid biased resulting from measurement error, we deleted values that were above or below four standard deviations of the mean. Before running the statistical analyses, we log-transformed non-normally distributed measures (leucocytes, HbA1c, glucose, HDL cholesterol, triglycerides, alanine transaminase (ALAT), aspartate transaminase (ASAT), alkaline phosphatase, gamma-glutamyl transferase (gamma-GT), serum creatinine, creatinine clearance, urine albumin, uric acid, thyroid stimulating hormone (TSH), free- triiodothyronine (free-T3), free-thyroxine (free-T4), albumin-to-creatinine ratio (ACR), urinary albumin excretion (UAE). We standardised all continuous measures before performing statistical analyses.

### Covariates

Age, sex and educational attainment were obtained through the baseline questionnaire. We used educational attainment as a proxy for SES, as has been done previously and because education is more differentiating than income in the Dutch population [20, 21]. Education was measured by a self-report question for participants, ‘What is the highest level of education you have attained?’ Education year was defined as ‘no education (did not finish primary school)’ as 1 year, ‘primary education’ as 7 years, ‘lower or preparatory secondary vocational education’ or ‘junior general secondary education’ as 10 years, ‘secondary vocational education’ or ‘senior general secondary education’ as 13 years, and ‘higher vocational education’ or ‘university education’ as 20 years. Genotyping chip and eight genetic principal components (PCs) were added as additional covariates.

### Polygenic risk scores

DNA samples were genotyped using the Illumina Global Screening Array and Illumina CytoSNP12v2 array. After quality control, both genotyping datasets were imputed at the Sanger imputation server using the Haplotype Reference Consortium panel v1.1 [22]. Details of genotyping, quality control and imputation in Lifelines for both genotyping datasets have been published elsewhere [23, 24]. After exclusion of non-European individuals (determined by self-report, outlier analysis and population stratification), 36 305 individuals genotyped using the Illumina Global Screening Array and 14 463 using the Illumina CytoSNP12v2 array were included in our analyses.

The PGS was calculated to represent the cumulative effects of many common genetic variants. We built the PGS using the most recent, and most sufficiently powered, meta genome-wide association study (GWAS) of ADHD conducted in 38 691 ADHD cases and 186 843 controls of European ancestry [12]. Multiallelic single nucleotide polymorphisms (SNPs) and SNPs with ambiguous strands (A/T or C/G) were removed from ADHD GWAS summary results. Overlapping SNPs across GWAS results and Lifelines sample with minor allele frequency (MAF > 1%) and imputation quality (INFO > 0.8) were kept. To obtain an independent set of SNPs, an LD-driven clumping procedure was performed in PLINK (r^2^ < 0.1, 250 kb window) using the LD reference panel of 503 European samples from 1000 Genomes phase 3 [25]. For each individual, PGS were calculated by multiplying the risk allele dosages for each SNP by its respective weight (the log of the odds ratio) and then summing all SNPs in the score. Scores were constructed at 11 selected P-value thresholds (5e^−8^, 1e^−^^7^, 1e^−6^, 1e^−5^, 1e^−4^, 1e^−3^, 0.01, 0.05, 0.1, 0.5, 1) and standardised using z-score transformations. Finally, principal component analysis (PCA) was performed on these scores and the first principal component was extracted as the final PGS. This approach is called PGS-PCA approach, which avoids optimising the parameters to construct the PGS and has been shown to be an unbiased and powerful way to index polygenic risk [26]. The data that support the findings of this study can be obtained through the submission and approval of a scientific proposal via the Lifelines biobank. Further details can be found at https://www.lifelines-biobank.com/.

### Statistical analysis

We estimated associations between ADHD-PGS and outcomes using generalised estimating equations implemented in the R-package DrGEE [27], and adjusted the standard errors for the non-independence of family data (siblings/parents/children/partners) using a sandwich estimator. For binary outcomes, we used the logit link function and reported Odds Ratios (ORs) (with 95% Confidence Intervals [95%CIs]). For continuous outcomes, we used the linear link function and reported standardised beta values (with 95%CIs). To assess the predictive ability of the ADHD-PGS, we calculated R^2^ (linear models) and Nagelkerke pseudo-R^2^ (logistic models) to represent the percentage variance explained by ADHD-PGS for each outcome. Nagelkerke pseudo-R2 was estimated for the full model (all covariates including ADHD-PGS) and the null model (not including ADHD-PGS). Percentage variance explained by ADHD-PGS for each outcome was calculated as the difference between the two (ΔR2).

We used a family fixed effects approach (i.e., comparing full siblings from the same family) to adjust associations for shared familial factors (using the same R-package DrGEE). We ran these analyses for outcomes that showed significant association with ADHD-PGS in the main results. Siblings within a family were treated as a separate stratum, thereby removing influences from factors shared between full siblings from the same family (see Fig. 1 for visual representation of analytical sibling design) [28]. Such factors include correlations between the PGS and shared family-environmental factors (e.g., environmentally mediated genetic effects such as SES, lifestyle, diet, household chaos, neighbourhood), population structure and assortative mating [29]. We tested for significant differences between the ADHD-PGS coefficients in the within- and between-sibling tests, to formally test if the effect of the ADHD-PGS statistically significantly attenuated after adjusting for shared familial factors [30].

In secondary analyses, we stratified the main analyses by age at baseline (younger/older than 60 years), sex (male/female) and educational attainment (low/high) to explore if there are differences in the aetiological associations between ADHD and cardiometabolic outcomes based on these factors. Moderation effects (ADHD-PGS*age, sex and educational attainment, respectively) were tested by including an interaction term to the main analyses, in separate models. Low educational attainment was defined as maximum primary school, or completed lower or secondary schooling, and high educational attainment was defined as completed higher vocational schooling or university education [21, 31]. In sensitivity analyses, we re-ran the main analyses excluding individuals with self-reported ADHD. If the associations remained, it would suggest that increased risk of cardiometabolic outcomes in ADHD is present even in those with subclinical ADHD. We also re-ran the main analyses additionally adjusting for baseline BMI, to investigate if the associations between ADHD-PGS and specific cardiometabolic outcomes were primarily explained by higher BMI levels.

In the statistical models, we adjusted for age, sex, genotyping chip, eight genetic principal components (PCs) accounting for population stratification, and interactions between genotyping chip and each genetic PC. We obtained false-discovery rate (FDR) corrected p-values using the Benjamini–Hochberg method to safeguard against multiple testing of all main and secondary tests (324 tests).

## Results

A total of 50,768 individuals were included in the study (27,819 (59%) females, 18–90 years at baseline (mean age=44.36, standard deviation [SD] = 13.60) (Table 1 for descriptive statistics). Within the study sample, 17,692 were full siblings from 7920 families. 383 (0.82%) individuals reported having an ADHD diagnosis, and the ADHD-PGS explained 0.83% of variance in self-reported ADHD (OR = 1.36 [95%CI = 1.23, 1.51], P = 4.3e^−8^).

**Table 1:** Descriptive characteristics of study population.

	Full sample (*N* = 50,768)	Sibling subsample (*N* = 17,692)
Age in years at baseline (Mean [SD])	44.36 (13.60)	40.32 (12.86)
Females/Males (N [% Females])	27,819/19,372 (59%)	9267/6145 (60%)
Completion of higher education (N [%])	13,460 (26.51%)	4495 (25.41%)
Average age difference between sibling pairs (Mean [SD])	NA	4.91 (3.66)

*SD* standard deviation.

### Association between ADHD-PGS and cardiometabolic diseases

**Fig. 2:**
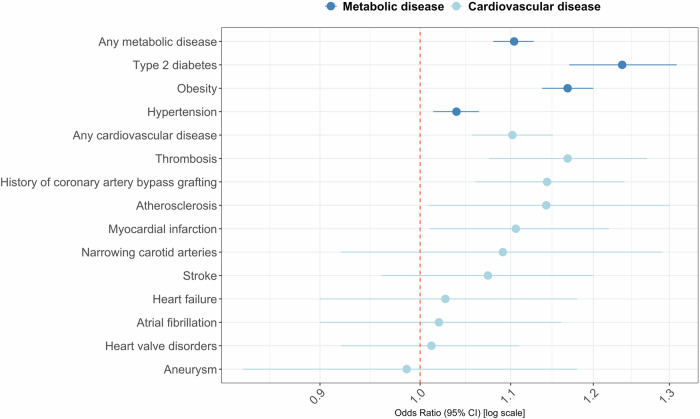
Associations between cardiometabolic diseases and ADHD-PGS (*N* = 50,768). Note: Plot of change in risk of each outcome associated with one standard deviation increase of the ADHD-PGS. Statistically significant associations with ADHD-PGS (adjusted p < 0.05): Any metabolic disease, Type 2 diabetes, Obesity, Hypertension, Any cardiovascular disease, Thrombosis, History of coronary artery bypass grafting.

The ADHD-PGS was statistically significantly associated with increased risk of developing any metabolic disease (OR = 1.10 [95%CI = 1.08, 1.13], R^2^ = 0.25%, P < 8.6e^−15^) and each separate metabolic disease: type-2 diabetes, obesity and hypertension (OR_range_=1.04–1.24, R^2^_range_ = 0.03–0.50%). The ADHD-PGS was also significantly associated with increased risk of having any cardiovascular disease (OR = 1.10 [95%CI = 1.06, 1.15], R^2^ = 0.12%, P = 9.0e^-5^). The ADHD-PGS was significantly associated with two of the nine specific cardiovascular diseases after adjustment for multiple testing: thrombosis and history of coronary artery bypass grafting (OR_range_=1.14–1.17, R^2^_range_ = 0.16–0.23%) (Fig. 2) (Table 2 for descriptive statistics, Supplementary Table 2 for full test estimates).

**Table 2:** Descriptive statistics on measures of ADHD, cardiometabolic diseases and biomarkers.

ADHD, cardiometabolic diseases and biomarkers	Sample size	Prevalence (%)
ADHD	50,768	383 (0.82)
Metabolic disease	50,768	14,133 (30.38)
Type 2 diabetes	50,768	1345 (2.66)
Obesity	50,768	6685 (13.21)
Hypertension	50,768	9621 (20.53)
Cardiovascular disease	50,768	2450 (5.19)
Thrombosis	50,768	598 (1.27)
History of coronary artery bypass grafting	50,768	658 (1.39)
Myocardial infarction	50,768	449 (0.95)
Atherosclerosis	50,768	226 (0.48)
Stroke	50,768	344 (0.73)
Narrowing carotid arteries	50,768	115 (0.24)
Heart failure	50,768	242 (0.51)
Atrial fibrillation	50,768	242 (0.51)
Heart valve disorders	50,768	492 (1.04)
Aneurysm	50,768	130 (0.28)
Cardiometabolic biomarker	Sample size	Mean (Standard deviation)
Glucose metabolic		
Glucose (mmol/L)	43,424	4.96 (0.73)
HbA1c (%)	43,411	5.49 (0.32)
Red blood cells		
Haematocrit (v/v)	46,985	0.42 (0.03)
Haemoglobin (mmol/L)	46,969	8.76 (0.76)
Lipid metabolism		
Total cholesterol (mmol/L)	47,052	5.09 (1.02)
LDL cholesterol (mmol/L)	47,055	3.25 (0.93)
HDL cholesterol (mmol/L)	47,039	1.47 (0.37)
Triglycerides (mmol/L)	46,723	1.11 (0.57)
Apolipo A1 in serum (g/L)	5245	1.61 (0.27)
Apolipo B100 (g/L)	5248	0.93 (0.24)
Liver function		
ALT (U/L)	23,750	22.18 (11.26)
AST (U/L)	23,790	23.88 (6.33)
Alkaline Phosphatase (U/L)	23,884	62.15 (16.71)
Gamma-GT (U/L)	23,734	24.44 (14.77)
Kidney function		
Creatinine (µmol/L)	46,997	73.29 (12.14)
Creatinine 24 h urine (mmol/L)	46,834	8.09 (3.74)
Estimated Glomerular Filtration Rate (eGFR)	47,088	99.85 (15.21)
Urinary albumin excretion (UAE) 24 h	23,902	11.53 (100.87)
Urinary albumin-creatinine ratio (UACR)	23,022	5.07 (47.02)
Uric acid (mmol/L)	58,470	0.29 (0.07)
Thyroid		
Free T3 (pmol/L)	13,875	5.22 (0.62)
Free T4 (pmol/L)	13,887	15.74 (2.05)
TSH (mU/L)	13,798	2.45 (1.51)
Inflammation		
hsCRP (mg/L)	22,781	2.90 (2.90)
Leucocytes (109/L)	46,872	5.98 (1.50)
Blood pressure		
Systolic BP	46,711	126.59 (16.56)
Diastolic BP	46,760	74.27 (10.08)

Sample size = Genotyped individuals with measured questionnaire or lab-based data. Values ± >4 SDs are excluded.

*BP* blood pressure, *hsCRP* high-sensitivity C-reactive protein, *TSH* Thyroid stimulating hormone, *Free T4* free thyroxine, *Free T3* free triiodothyronine, *Apolipo* Apolipoprotein, *Gamma-GT* Gamma-glutamyl transferase, *AST* Aspartate aminotransferase, *ALT* Alanine transaminase, *HDL* high-density lipoprotein, *LDL* low-density lipoprotein, *HbA1c* Haemoglobin A1c.

### Association between ADHD-PGS and cardiometabolic biomarkers

**Fig. 3:**
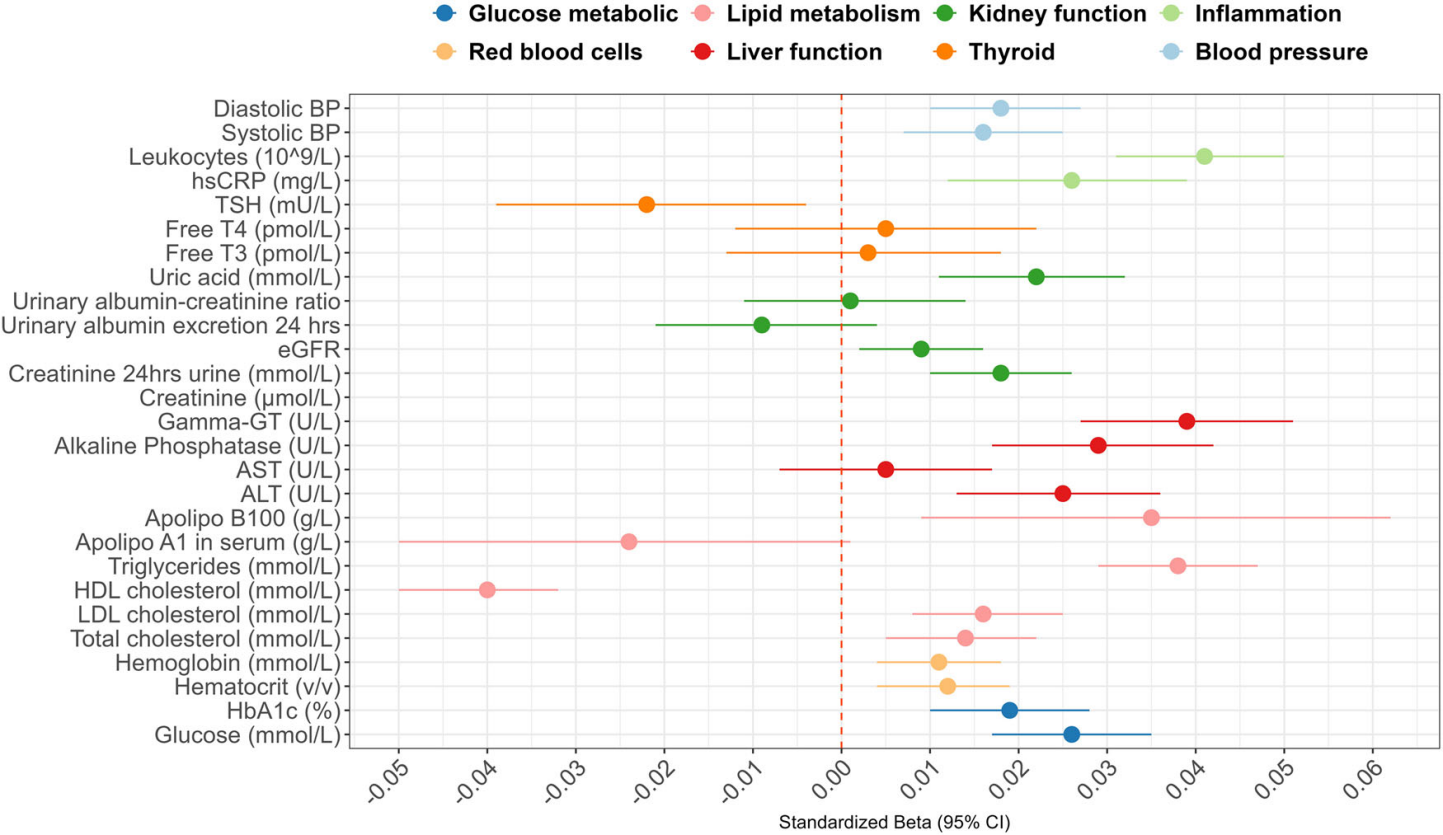
Associations between cardiometabolic biomarkers and ADHD-PGS (*N* = 47 189). Note: Plot of change in standard deviation of each biomarker associated with one standard deviation increase of the ADHD-PGS. BP Blood pressure, hsCRP High-sensitivity C-reactive protein, Free T4 Free thyroxine, Free T3 Free triiodothyronine, eGFR Estimated glomerular filtration rate, Apolipo B100 Apolipoprotein B100, Gamma-GT Gamma-glutamyl transferase, AST Aspartate aminotransferase, ALT Alanine transaminase, HDL High-density lipoprotein, LDL Low-density lipoprotein, HbA1c Haemoglobin A1c. Statistically significant associations with ADHD-PGS (adjusted p < 0.05): Glucose, HbA1c, Haematocrit, Hemaglobin, Total/LDL/HDL cholesterol, Triglycerides, Apolipo B100, ALT, Alkaline phosphatase, Gamma-GT, Creatinine, Creatinine 24 h urine, Uric acid, hsCRP, Leucocytes, Systolic/Diastolic blood pressure.

The ADHD-PGS was statistically significantly associated with 19 of the 27 biomarkers (*P* < 0.05), after correction for multiple testing. Associations were statistically significant for measures across the different functional groups: glucose metabolic (Beta_range _= 0.02–0.03, R^2^_range_ = 0.03–0.07%), red blood cells (Beta = 0.01, R^2^ = 0.01%), lipid metabolism (Beta_range_ = −0.04–0.04, R^2^_range_ = 0.02–0.16%), liver function (Beta_range _= 0.03–0.04, R^2^_range_ = 0.06–0.15%), kidney function (Beta_range_ = −0.01–0.02, R^2^_range_ = 0.01–0.05%), inflammation (Beta_range _= 0.03–0.04, R^2^_range_ = 0.08–0.16%) and blood pressure (Beta = 0.02, R^2^ = 0.03%) (Fig. 3) (Supplementary Table 3 for estimates).

### Within-sibling analyses

We first re-ran the main analyses in the sibling subsample (between-siblings) (*N* = 17,692) to create a reference for the within-sibling analyses (Supplementary Table 4). This ensured that any attenuation of associations in the within-sibling analyses were not mainly due to loss of statistical power or any difference in study population. In the sibling subsample, 12 cardiometabolic outcomes remained significantly associated with ADHD-PGS. The ADHD-PGS was still significantly associated with having any metabolic disease (OR = 1.11 [95%CI = 1.07, 1.16], P = 4.38e-6) but not any cardiovascular disease (*P* = 0.50). We did not re-run analyses for the distinct metabolic and cardiovascular diseases due to the low sample sizes of these subgroups of siblings with distinct diseases.

In the within-sibling analyses, when comparing full siblings, there only remained one statistically significant association with ADHD-PGS; namely metabolic disease (OR = 1.11 [95%CI = 1.02, 1.20], P = 0.03) (Table 3). Overall, when adjusting for shared familial factors, the PGS-outcome association effect sizes attenuated on average 56%. These within-sibling effect size estimates were statistically significantly different from estimates in the between-sibling analyses for metabolic disease and nine (out of 19) of the biomarker measures (*P* < 0.05).

**Table 3:** Within-sibling associations between cardiometabolic outcomes and ADHD-PGS (unique individuals *N* = 17 692).

Condition	Prev. (%) / Mean (SD)	OR/Beta (95%CI)	% change log(OR)/Beta	P_between-within sib_
Metabolic disease	3926 (25.47%)	**1.108 (1.023, 1.199)**^**a**^	−1.72	**0.03**^**a**^
Cardiovascular disease	556 (3.58%)	1.055 (0.879, 1.266)	+19.06	0.31
Biomarkers				
Glucose (mmol/L)	4.86 (0.50)	−0.005 (−0.031, 0.020)	−129.41	0.90
HbA1c (%)	5.47 (0.31)	−0.005 (−0.029, 0.019)	−50.00	**0.02**^**a**^
Haematocrit (v/v)	0.42 (0.03)	0.015 (−0.006, 0.036)	−21.05	0.15
Haemoglobin (mmol/L)	8.76 (0.77)	0.016 (−0.005, 0.036)	−11.11	**0.05**^**a**^
Total cholesterol (mmol/L)	5.02 (1.01)	0.001 (−0.022, 0.025)	−93.33	0.08
LDL cholesterol (mmol/L)	3.19 (0.93)	−0.001 (−0.024, 0.022)	−105.88	0.26
HDL cholesterol (mmol/L)	1.48 (0.37)	−0.022 (−0.045, 0.001)	−40.54	**7.30e-8**^**a**^
Triglycerides (mmol/L)	1.08 (0.56)	0.022 (−0.003, 0.048)	−42.11	**7.36e-3**^**a**^
Apolipo B100 (g/L)	0.92 (0.24)	0.032 (−1.017, 0.165)	−25.71	0.96
ALT (U/L)	21.86 (11.16)	0.002 (−0.039, 0.043)	−88.23	0.66
Alkaline Phosphatase (U/L)	61.36 (16.57)	0.018 (−0.023, 0.059)	−48.57	0.15
Gamma-GT (U/L)	23.79 (14.37)	0.026 (−0.015, 0.066)	−36.58	**0.02**^**a**^
Creatinine (µmol/L)	72.90 (11.87)	−0.011 (−0.031, 0.009)	−31.25	**9.12e-3**^**a**^
Creatinine 24 h urine (mmol/L)	8.23 (3.78)	0.023 (−0.001, 0.047)	−8.00	**2.99e-8**^**a**^
Uric acid (mmol/L)	0.29 (0.07)	−0.001 (−0.036, 0.033)	−105.26	0.65
hsCRP (mg/L)	2.08 (2.45)	−0.011 (−0.062, 0.039)	−142.31	0.11
Leucocytes (10^9/L)	5.97 (1.50)	0.031 (0.004, 0.058)	−22.50	**7.30e-8**^**a**^
Systolic BP	124.96 (15.69)	−0.008 (−0.031, 0.015)	−140.00	0.31
Diastolic BP	73.39 (9.95)	0.003 (−0.021, 0.028)	−87.50	0.85

Fixed effects models controlling for genotyping chip, 8 PCs, PC*chip, sex, age.

Average attenuation in within-sibling (compared to between-sibling) models for cardiometabolic measures: −56%.

The within-sibling analyses are performed for outcomes that showed significant association with ADHD-PGS in main analyses (Figs. 1, 2). Did not include apolipoproteins due to low sample sizes.

*BP* Blood pressure, *hsCRP* High-sensitivity C-reactive protein, *Apolipo* Apolipoprotein, *Gamma-GT* Gamma-glutamyl transferase, *ALT* Alanine transaminase, *HDL* high-density lipoprotein, *LDL* low-density lipoprotein, *HbA1c* Haemoglobin A1c.

^a^Statistically significant (*p* < 0.05) FDR-adjusted *p* value. P_between-within sib_ = statistical comparison of ADHD-PGS estimate in between-sibling versus within-sibling model.

### Sensitivity analyses

The main results did not change in terms of effect sizes and statistical significance when we excluded individuals with self-reported ADHD (Supplementary Tables 5, 6). When the main analyses were additionally adjusted for baseline BMI levels, all the associations with cardiometabolic diseases remained statistically significant except for the association between ADHD-PGS and hypertension (*P* = 0.89). Most of the associations with cardiometabolic biomarkers, however, were no longer statistically significant after adjusting for BMI, with the exception of HDL cholesterol, triglycerides, gamma-GT, creatinine, TSH levels and leucocytes (Supplementary Tables 7, 8).

### Analyses stratified by sex, age and SES

The associations between ADHD-PGS and cardiometabolic outcomes were overall similar across the sex, age and educational attainment groups, and very few moderation effects (of sex, age and educational attainment) with ADHD-PGS were statistically significant (Supplementary Tables 9-14).

## Discussion

In this large-scale population study, we showed that polygenic load for ADHD, based on the latest meta-GWAS [12], was significantly associated with poorer cardiometabolic health, as indexed by metabolic and cardiovascular diseases, as well as a key biomarkers. Adjustment for familial factors shared by siblings attenuated most of the associations between ADHD-PGS and cardiometabolic outcomes (on average 56% attenuated effect sizes) and only metabolic disease remained statistically significant. Associations were generally similar across age, sex and SES.

One SD increase in the ADHD-PGS was associated with a 10% higher risk of developing any metabolic and any cardiovascular disease, respectively. The specific disorders that were significantly associated with ADHD-PGS were type-2 diabetes, obesity, hypertension, thrombosis and history of coronary artery bypass grafting. These findings are consistent with recent research findings on phenotypic and genetic links between ADHD and metabolic disease, peripheral vascular disease, ischaemic heart disease and heart failure [6, 8, 12]. We further extended previous research by showing that ADHD-PGS was significantly associated with numerous cardiometabolic biomarkers. The strongest associations were seen for measures related to lipid metabolism, liver function and inflammation. For example, a one SD increase in the ADHD-PGS was associated with a 0.04 SD lower HDL cholesterol (mmol/L) value (ADHD-PGS explained 0.16% of variance in HDL cholesterol). The directions of effects largely implicate poorer biological health profiles in those with higher polygenic load for ADHD. While there is very limited research investigating biomarker measures in individuals with ADHD, mostly consisting of small, clinical paediatric studies (where the effect of ADHD medication may influence results), our results are in line with past findings implicating more inflammation, more detrimental lipid levels and glucose dysregulation in individuals with ADHD compared to those without [32–35]. Our results also confirmed, and extended, findings from the latest meta-GWAS on ADHD reporting genetic correlations between ADHD and higher levels of HbA1c, triglycerides, urate and hypertension and lower levels of HDL cholesterol [12]. Our results further showed that BMI levels largely explained the associations between ADHD-PGS and several blood-based cardiometabolic biomarkers, specifically relating to glucose metabolism, LDL cholesterol, blood pressure and certain measures of kidney and liver function. As BMI is a modifiable risk factor, our findings strongly suggest that weight loss may be a highly effective target for prevention and intervention strategies for cardiometabolic health issues in individuals with ADHD. Overall, our findings warrant further investigation into the specific biological mechanisms that are implicated in adult ADHD, and which may explain the link with cardiometabolic events.

An important finding is that polygenic load for ADHD was associated with cardiometabolic outcomes in the general population, even when individuals with self-reported ADHD were excluded. This suggests that the increased risks of cardiometabolic diseases and poor health indicators in ADHD may not be driven by negative effects from clinically diagnosed ADHD, such as medication treatment and may be present in those with subclinical ADHD and higher ADHD genetic burden. While these conclusions should be cautiously considered in light of the relatively low predictive ability of the ADHD-PGS on cardiometabolic outcomes, they are consistent with recent genetic and epidemiological evidence [7, 13, 36]. These recent studies have confirmed the associations between ADHD and cardiometabolic outcomes in unmedicated populations [7, 13], and shown that elevated ADHD symptoms increase the risk for cardiometabolic disorders in the general population [36]. This is important to highlight given that ADHD medication rates are high in clinical populations [37], and it has been suggested that ADHD medications may confer increased risk of health outcomes, such as cardiovascular disease, although evidence for this is limited [38].

We found that the PGS-cardiometabolic associations were largely attenuated by shared familial factors. These attenuations were generally consistent across outcomes, and were statistically significant for metabolic disease and several biomarker measures, suggesting influence from shared family-environmental factors. Such family-environmental factors may include the association between ADHD and lower parental SES (income, educational attainment, neighbourhood SES) or poor lifestyle (relating to diet and physical activity), which may be elevated when a parent has ADHD and are associated with higher risk of cardiometabolic diseases [29, 39–42]. Future studies should study specific family-environmental factors in further detail, to inform targeted interventions for cardiometabolic disease in families of individuals with ADHD. It is also important to note that the association with self-reported metabolic disease did not attenuate and remained significant, suggesting that the increased risk of metabolic disease may be to some extent directly driven by an individuals’ polygenic load for ADHD than via family-environmental factors, to a larger extent than cardiovascular diseases. Mendelian randomisation studies could be used to further investigate to what extent this association is causal, rather than explained by pleiotropic effects. Indeed, preliminary results from a recent mendelian randomisation study found support for a causal role of ADHD on childhood obesity [43].

Within-sibling PGS analyses adjust for similar environments shared by siblings (indirect parental genetic effects) as well as population stratification and assortative mating, which can bias PGS effects in non-sibling models. Thus, it is thought that sibling analyses can estimate direct genetic effects. In support of this method, a study comparing indirect parental genetic effects of PGS on educational outcomes found consistent results across adoption, parent-offspring and sibling designs—each with their own sets of limitations and potential bias [44]. The study findings suggested that parental cognitive and non-cognitive skills influenced offspring education through the environment, with indirect genetic effects explained 36–40% of population PGS associations [44]. However, it has recently been highlighted that PGS sibling-comparison results should be interpreted with caution [45], as the models may introduce other types of bias, such as from correlations between (non-genetically influenced) environmental factors affecting the two siblings, and as the models rely on assumptions that direct and indirect effects are easily separable [45]. Further research is needed to investigate the direct and indirect genetic effects of the ADHD-PGS on cardiometabolic outcomes using triangulation with different study designs, and more detailed investigations into specific environmental factors that mediate parent PGS effects.

We did not find strong evidence for differences in the ADHD-PGS associations as a function of age, sex, or SES. Previous PGS studies examining genetic associations between ADHD and health outcomes also failed to find strong evidence for moderation effects of educational attainment and sex, based on the previous ADHD GWAS [13, 46], and a recent large-scale family-based register study also did not find support for sex differences [6]. While these results suggest that there are no substantial sex, age and SES moderation effects of the genetic associations between ADHD and cardiometabolic outcomes, it is challenging to detect interaction effects using PGS as it requires large and well-powered studies. Further, disorder PGSs may not necessarily capture the genetic variants linked to a differential susceptibility to risk-factors exposure. This could potentially explain why we did not find strong evidence for moderation effects in this study.

### Strengths and limitations

The strengths of this study include the use of a large population-based cohort study, with rich phenotypic, genetic and family-level data. Our PGS was built on the most recent GWAS on ADHD, which is considerably more well-powered than the previous GWAS [12].

Limitations include the low predictive ability of the ADHD-PGS, which has previously been shown to explain up to 5.5% of the variance in ADHD case-control status [47]. This should be considered when interpreting the results, as a lack of statistical significance for specific outcomes does not necessarily mean that there is no true association with the ADHD-PGS. The low predictive ability is especially an issue in the stratified analyses, where the sample sizes were smaller and statistical power lower than in the main analyses. However, we have emphasised the general trends in results from the secondary analyses rather than focused on significance testing of specific outcomes. Furthermore, ADHD-PGS only captures part of the ADHD heritability [12], thus, an absence of an associations does not mean that there is definitely no association or moderation effect. Even larger studies and more powerful ADHD-PGS will be needed in future studies to more precisely confirm and further interpret findings. Second, we used self-report measures for several disease outcomes, which includes more subjective (e.g., recall) bias than clinical diagnoses, and may have led to underestimations of the cardiometabolic diseases. While this is important to consider when interpreting our results, a recent study showed that ADHD-PGS associations with cardiometabolic diseases were overall similar across definitions using self-report and clinical diagnoses, supporting the general reliability of our findings [13]. Specifically with regard to the secondary analyses in which we removed individuals with ADHD, underestimation not only results from recall problems but also from underdiagnosis of ADHD in adulthood. That is, individuals reported on clinically diagnosed ADHD and it is likely that not all adults with ADHD were left out of these analyses. Another potential limitation to highlight in terms of cardiometabolic outcomes is that we adjusted specific biomarker measures based on use of medications that target those measures. However, the medications may have wider impact beyond the target biomarkers. For example, cholesterol-lowering medication may have indirect impact on apolipoprotein B100, and blood pressure-lowering medications on renal function [48, 49]. While we used standard procedures to adjust for medication effects, as has been done in previous research [18, 19], it is important to interpret results with caution and consider that other biomarkers may have been influenced by medication use. Furthermore, individuals in the Lifelines study have been found to have a somewhat higher SES and be slightly healthier than the general population. However, a comparison study using the Dutch Population Register showed that the Lifelines cohort was broadly representative of the adult population of the northern part of Netherlands [50]. Higher ADHD genetic liability has further been negatively associated with study participation [51]. Therefore, any observed associations were likely attenuated compared to those in the general population and in clinical ADHD samples. Finally, we only included individuals with European ancestry in these genetic analyses, which will limit generalisability to more diverse populations.

## Conclusion

Our findings suggest that higher ADHD genetic liability is linked to several metabolic and cardiovascular diseases and biomarkers in adults. These genetic associations were observable in the general population, even in the absence of individuals with ADHD and were in part explained by indirect effects via familial factors shared by siblings.

## Supplementary information

Supplementary material

## Data Availability

The data that support the findings of this study can be obtained through the submission and approval of a scientific proposal via the Lifelines biobank. Further details can be found at https://www.lifelines-biobank.com/.

## Code availability

Statistical analyses were performed in R (v.4.2.2). The codes are available upon request.
