# Supplementary material for: The contribution of attention-deficit/hyperactivity disorder polygenic load to metabolic and cardiovascular health outcomes: a large-scale population and sibling study
Source: Transl Psychiatry. 2024 Nov 13;14:470. doi: 10.1038/s41398-024-03178-2 (PMC11561358; doi:10.1038/s41398-024-03178-2)
Supplement: Supplementary file 1 — Supplementary material [file 41398_2024_3178_MOESM1_ESM.docx]

**Supplemental information:**

**The contribution of attention-deficit/hyperactivity disorder polygenic load to metabolic and cardiovascular health outcomes: a large-scale population and sibling study**

Ebba Du Rietz, PhD, Tian Xie, PhD, Rujia Wang, PhD, Rosa Cheesman, PhD, Miguel Garcia-Argibay, PhD, Zihan Dong, MSc, Jia Zhang, Jacobien Niebuur, PhD, Melissa Vos, PhD, Harold Snieder, PhD, Henrik Larsson, PhD, Catharina A. Hartman

Table of Contents

[Supplementary Figure 1. Flow chart of genetic data processing 2](#_Toc163829488)

[Supplementary Table 1. Definitions of cardiometabolic diseases 3](#_Toc163829489)

[Supplementary Table 2. Associations of the ADHD-PGS with cardiometabolic diseases (N=50 768) 4](#_Toc163829490)

[Supplementary Table 3. Associations between cardiometabolic biomarkers and ADHD-PGS (N=47 189) 5](#_Toc163829491)

[Supplementary Table 4. Associations between cardiometabolic outcomes and ADHD-PGS in sibling cohort (unique individuals N=17 692) 7](#_Toc163829492)

[Supplementary Table 5. Associations between cardiometabolic outcomes and ADHD-PGS: excluding individuals with self-reported ADHD (N=50 385) 8](#_Toc163829493)

[Supplementary Table 6. Associations between cardiometabolic biomarkers and ADHD-PGS: excluding individuals with self-reported ADHD (N=46 806) 9](#_Toc163829494)

[Supplementary Table 7. Associations between cardiometabolic outcomes and ADHD-PGS: additionally adjusting for BMI 10](#_Toc163829495)

[Supplementary Table 8. Associations between cardiometabolic biomarkers and ADHD-PGS: additionally adjusting for BMI 11](#_Toc163829496)

[Supplementary Table 9. Associations between cardiometabolic diseases and ADHD-PGS: stratified by sex 12](#_Toc163829497)

[Supplementary Table 10. Associations between cardiometabolic biological risk indicators and ADHD-PGS: stratified by sex 13](#_Toc163829498)

[Supplementary Table 11. Associations between cardiometabolic diseases and ADHD-PGS: stratified by age group 15](#_Toc163829499)

[Supplementary Table 12. Associations between cardiometabolic biological risk indicators and ADHD-PGS: stratified by age group 16](#_Toc163829500)

[Supplementary Table 13. Associations between cardiometabolic diseases and ADHD-PGS: stratified by educational attainment 18](#_Toc163829501)

[Supplementary Table 14. Associations between cardiometabolic biological risk indicators and ADHD-PGS: stratified by educational attainment 19](#_Toc163829502)

# Supplementary Figure 1. Flow chart of genetic data processing

34 removed due to non-European ancestry

3,324 removed after quality control

50,768 participants

50,802 participants

54,126 participants with genetic data

# Supplementary Table 1. Definitions of cardiometabolic diseases

| **Cardiometabolic disease** | **Definition** |
| --- | --- |
| ***Metabolic disease*** |  |
| Type 2 diabetes | Self-reported diabetes (excluding cases with type 1 or other type of diabetes)  **AND**  (self-reported diabetes medication use (ATC codes A10 to A10X) or HbA1c>=6.5%  **OR**  fasting plasma glucose is >=7.0 mmol/L) |
| Obesity | Body mass index (kg/m2 >=30), calculated from weight and height measured at physical examination |
| Hypertension | Self-reported hypertension |
| **Cardiovascular disease** |  |
| Thrombosis | Self-reported |
| History of coronary artery bypass (CABG) | Self-reported |
| Myocardial infarction | Self-reported myocardial infarction  **AND**  Drug use related to myocardial infarction or ECG pathology |
| Atherosclerosis | Self-reported |
| Stroke | Self-reported |
| Narrowing carotid arteries | Self-reported |
| Heart failure | Self-reported heart failure  **AND**  Drug use related to heart failure or Pacemaker/ICD implantation or Heart transplantation |
| Atrial fibrillation | Self-reported atrial fibrillation and use of vit K antagonist or NOAC  **OR**  Atrial fibrillation on ECG and use of vit K antagonist  **OR**  CHADVASC > 2 and atrial fibrillation on ECG |
| Heart valve disorders | Self-reported |
| Aneurysm | Self-reported |

ATC: Anatomical Therapeutic Chemical code, HbA1c: Hemoglobin A1c, ECG: Electrocardiogram, CHADVASC: CHA2DS2-VASc Calculator for Atrial Fibrillation, NOAC: Novel oral anticoagulants

# Supplementary Table 2. Associations of the ADHD-PGS with cardiometabolic diseases (N=50 768)

| **Condition** | **Prevalence (%)** | **OR (95% CI)** | **R^2^ (%)** | **P_adj_ value** |
| --- | --- | --- | --- | --- |
| ***ADHD*** | 383 (0.82) | 1.362 (1.231, 1.507) | 0.83% | **4.33e-8** |
| ***Metabolic disease*** | 14 133 (30.38) | 1.104 (1.080, 1.127) | 0.25% | **8.60e-15** |
| Type 2 diabetes | 1 345 (2.66) | 1.237 (1.169, 1.308) | 0.50% | **3.19e-12** |
| Obesity | 6 685 (13.21) | 1.168 (1.137, 1.200) | 0.50% | **8.60e-15** |
| Hypertension | 9 621 (20.53) | 1.039 (1.014, 1.064) | 0.03% | **1.06e-2** |
| ***Cardiovascular disease*** | 2 450 (5.19) | 1.102 (1.056, 1.150) | 0.12% | **9.00e-5** |
| Thrombosis | 598 (1.27) | 1.168 (1.075, 1.269) | 0.23% | **5.47e-3** |
| History of coronary artery bypass grafting | 658 (1.39) | 1.143 (1.056, 1.238) | 0.16% | **5.47e-3** |
| Myocardial infarction | 449 (0.95) | 1.106 (1.006, 1.216) | 0.09% | 0.11 |
| Atherosclerosis | 226 (0.48) | 1.142 (1.008, 1.295) | 0.14% | 0.11 |
| Stroke | 344 (0.73) | 1.074 (0.962, 1.199) | 0.04% | 0.38 |
| Narrowing carotid arteries | 115 (0.24) | 1.091 (0.923, 1.290) | 0.05% | 0.50 |
| Heart failure | 242 (0.51) | 1.027 (0.898, 1.175) | 0.01% | 0.85 |
| Atrial fibrillation | 242 (0.51) | 1.020 (0.897, 1.160) | 0.003% | 0.87 |
| Heart valve disorders | 492 (1.04) | 1.012 (0.921, 1.112) | 0.001% | 0.89 |
| Aneurysm | 130 (0.28) | 0.986 (0.828, 1.175) | 0.001% | 0.94 |

Note: Analyses adjusting for genotyping chip, 8 PCs, PC*chip, sex, age, and cluster by fam_ID (children, parents, siblings, partners). Obesity: BMI > 30.

P_adj_ = FDR-adjusted p values, statistically significant in bold

# Supplementary Table 3. Associations between cardiometabolic biomarkers and ADHD-PGS (N=47 189)

| **Measure** | **N** | **Mean (SD)** | **Standardized beta**  **(95% CI)** | **R^2^ (%)** | **P_adj_ value** |
| --- | --- | --- | --- | --- | --- |
| ***Glucose metabolic*** |  |  |  |  |  |
| Glucose (mmol/L) | 43 424 | 4.96 (0.73) | 0.026 (0.017, 0.035) | 0.07% | **1.84e-7** |
| HbA1c (%) | 43 411 | 5.49 (0.32) | 0.019 (0.010, 0.028) | 0.03% | **3.62e-4** |
| ***Red blood cells*** |  |  |  |  |  |
| Hematocrit (v/v) | 46 985 | 0.42 (0.03) | 0.012 (0.004, 0.019) | 0.01% | **0.01** |
| Hemoglobin (mmol/L) | 46 969 | 8.76 (0.76) | 0.011 (0.004, 0.018) | 0.01% | **0.01** |
| ***Lipid metabolism*** |  |  |  |  |  |
| Total cholesterol (mmol/L) | 47 052 | 5.09 (1.02) | 0.014 (0.005, 0.022) | 0.02% | **1.31e-3** |
| LDL cholesterol (mmol/L) | 47 055 | 3.25 (0.93) | 0.016 (0.008, 0.025) | 0.03% | **1.31e-3** |
| HDL cholesterol (mmol/L) | 47 039 | 1.47 (0.37) | -0.040 (-0.050, -0.032) | 0.16% | **8.60e-15** |
| Triglycerides (mmol/L) | 46 723 | 1.11 (0.57) | 0.038 (0.029, 0.047) | 0.14% | **2.09e-14** |
| Apolipo A1 in serum (g/L) | 5 245 | 1.61 (0.27) | -0.024 (-0.050, 0.001) | 0.06% | 0.15 |
| Apolipo B100 (g/L) | 5 248 | 0.93 (0.24) | 0.035 (0.009, 0.062) | 0.13% | **0.03** |
| ***Liver function*** |  |  |  |  |  |
| ALT (U/L) | 23 750 | 22.18 (11.26) | 0.025 (0.013, 0.036) | 0.06% | **2.22e-4** |
| AST (U/L) | 23 790 | 23.88 (6.33) | 0.005 (-0.007, 0.017) | 0.01% | 0.64 |
| Alkaline Phosphatase (U/L) | 23 884 | 62.15 (16.71) | 0.029 (0.017, 0.042) | 0.09% | **5.88e-5** |
| Gamma-GT (U/L) | 23 734 | 24.44 (14.77) | 0.039 (0.027, 0.051) | 0.15% | **1.64e-9** |
| ***Kidney function*** |  |  |  |  |  |
| Creatinine (µmol/L) | 46 997 | 73.29 (12.14) | -0.011 (-0.019, -0.003) | 0.01% | **0.02** |
| Creatinine 24hrs urine (mmol/L) | 46 834 | 8.09 (3.74) | 0.018 (0.010, 0.026) | 0.03% | **6.98e-5** |
| Estimated Glomerular Filtration Rate (eGFR) | 47 088 | 99.85 (15.21) | 0.009 (0.002, 0.016) | 0.01% | 0.07 |
| Urinary albumin excretion (UAE) 24 hrs | 23 902 | 11.53 (100.87) | -0.009 (-0.021, 0.004) | 0.01% | 0.33 |
| Urinary albumin-creatinine ratio (UACR) | 23 022 | 5.07 (47.02) | 0.001 (-0.011, 0.014) | <0.001% | 0.90 |
| Uric acid (mmol/L) | 58 470 | 0.29 (0.07) | 0.022 (0.011, 0.032) | 0.05% | **3.76e-4** |
| ***Thyroid*** |  |  |  |  |  |
| Free T3 (pmol/L) | 13 875 | 5.22 (0.62) | 0.003 (-0.013, 0.018) | <0.001% | 0.87 |
| Free T4 (pmol/L) | 13 887 | 15.74 (2.05) | 0.005 (-0.012, 0.022) | 0.01% | 0.74 |
| TSH (mU/L) | 13 798 | 2.45 (1.51) | -0.022 (-0.039, -0.004) | 0.05% | 0.06 |
| ***Inflammation*** |  |  |  |  |  |
| hsCRP (mg/L) | 22 781 | 2.90 (2.90) | 0.026 (0.012, 0.039) | 0.08% | **1.31e-3** |
| Leukocytes (10^9/L) | 46 872 | 5.98 (1.50) | 0.041 (0.031, 0.050) | 0.16% | **8.60e-15** |
| ***Blood pressure*** |  |  |  |  |  |
| Systolic BP | 46 711 | 126.59 (16.56) | 0.016 (0.007, 0.025) | 0.03% | **1.31e-3** |
| Diastolic BP | 46 760 | 74.27 (10.08) | 0.018 (0.010, 0.027) | 0.03% | **8.60e-15** |

Note: Analyses adjusting for genotyping chip, 8 PCs, PC*chip, sex, age, and cluster by fam_ID (children, parents, siblings, partners). Values > 4 SDs are excluded. P_adj_ = FDR-adjusted p values, statistically significant in bold.

# Supplementary Table 4. Associations between cardiometabolic outcomes and ADHD-PGS in sibling cohort (unique individuals N=17 692)

| **Condition** | **OR/Beta (95% CI)** | **P_adj_ value** |
| --- | --- | --- |
| **Metabolic disease** | 1.110 (1.067, 1.155) | **4.38e-6** |
| **Cardiovascular disease** | 1.046 (0.957, 1.143) | 0.50 |
| **Biomarkers** |  |  |
| Glucose (mmol/L) | 0.017 (0.001, 0.032) | 0.11 |
| HbA1c (%) | -0.010 (-0.006, 0.025) | 0.42 |
| Hematocrit (v/v) | 0.019 (0.006, 0.032) | **0.02** |
| Hemoglobin (mmol/L) | 0.018 (0.005, 0.031) | **0.02** |
| Total cholesterol (mmol/L) | 0.015 (-0.001, 0.030) | 0.15 |
| LDL cholesterol (mmol/L) | 0.017 (0.002, 0.033) | 0.08 |
| HDL cholesterol (mmol/L) | -0.037 (-0.053, -0.022) | **4.52e-5** |
| Triglycerides (mmol/L) | 0.038 (0.022, 0.054) | **5.81e-5** |
| ALT (U/L) | 0.017 (-0.005, 0.039) | 0.26 |
| Alkaline Phosphatase (U/L) | 0.035 (0.011, 0.058) | **0.02** |
| Apolipo B100 (g/L) | 0.031 (-0.029, 0.091) | 0.50 |
| Gamma-GT (U/L) | 0.041 (0.019, 0.063) | **5.47e-3** |
| Creatinine (µmol/L) | -0.016 (-0.030, -0.003) | **0.07** |
| Creatinine 24hrs urine (mmol/L) | 0.025 (0.010, 0.039) | **5.47e-3** |
| Uric acid (mmol/L) | 0.019 (-0.003, 0.038) | 0.14 |
| hsCRP (mg/L) | 0.026 (0.001, 0.052) | 0.13 |
| Leukocytes (10^9/L) | 0.040 (0.023, 0.058) | **4.94e-5** |
| Systolic BP | 0.020 (0.005, 0.034) | **0.03** |
| Diastolic BP | 0.024 (0.008, 0.039) | **0.01** |

Note: Analyses adjusting for genotyping chip, 8 PCs, PC*chip, sex, age, and cluster by SIB_ID.

Values > 4 SDs are excluded. Only including outcomes that were significantly associated with ADHD-PGS in the main analysis in the full cohort. P_adj_ = FDR-adjusted p values, statistically significant in bold.

# Supplementary Table 5. Associations between cardiometabolic outcomes and ADHD-PGS: excluding individuals with self-reported ADHD (N=50 385)

| **Condition** | **OR (95% CI)** | **P_adj_ value** |
| --- | --- | --- |
| ***Metabolic disease*** | 1.103 (1.080, 1.127) | **8.60e-15** |
| Type 2 diabetes | 1.239 (1.171, 1.311) | **2.64e-12** |
| Obesity | 1.167 (1.136, 1.200) | **8.50e-15** |
| Hypertension | 1.037 (1.012, 1.062) | **0.01** |
| ***Cardiovascular disease*** | 1.102 (1.056, 1.150) | **1.01e-4** |
| Thrombosis | 1.167 (1.074, 1.269) | **5.47e-3** |
| History of coronary artery bypass grafting (CABG) | 1.143 (1.055, 1.238) | **5.47e-3** |
| MI | 1.107 (1.007, 1.217) | 0.11 |
| Atherosclerosis | 1.140 (1.005, 1.294) | 0.11 |
| Stroke | 1.068 (0.956, 1.193) | 0.42 |
| Narrowing carotid arteries | 1.091 (0.920, 1.294) | 0.50 |
| Heart failure | 1.022 (0.893, 1.169) | 0.87 |
| Atrial fibrillation | 1.021 (0.898, 1.161) | 0.87 |
| Heart valve disorders | 1.016 (0.924, 1.117) | 0.87 |
| Aneurysm | 0.987 (0.829, 1.176) | 0.95 |

Note: Adults with self-reported ADHD were excluded from these analyses (N=383).

P_adj_ = FDR-adjusted p values, statistically significant in bold.

# Supplementary Table 6. Associations between cardiometabolic biomarkers and ADHD-PGS: excluding individuals with self-reported ADHD (N=46 806)

| **Measure** | **Standardized beta (95% CI)** | **P_adj_ value** |
| --- | --- | --- |
| ***Glucose metabolic*** |  |  |
| Glucose (mmol/L) | 0.026 (0.017, 0.035) | **1.88e-7** |
| HbA1c (%) | 0.019 (0.010, 0.028) | **4.02e-4** |
| ***Red blood cells*** |  |  |
| Hematocrit (v/v) | 0.011 (0.004, 0.018) | **0.02** |
| Hemoglobin (mmol/L) | 0.010 (0.003, 0.017) | **0.02** |
| ***Lipid metabolism*** |  |  |
| Total cholesterol (mmol/L) | 0.014 (0.005, 0.022) | **1.88e-7** |
| LDL cholesterol (mmol/L) | 0.016 (0.008, 0.025) | **4.02e-4** |
| HDL cholesterol (mmol/L) | -0.040 (-0.049, -0.031) | **1.88e-7** |
| Triglycerides (mmol/L) | 0.038 (0.028, 0.047) | **4.02e-4** |
| Apolipo A1 in serum (g/L) | -0.026 (-0.051, 0.001) | 0.12 |
| Apolipo B100 (g/L) | 0.033 (0.007, 0.060) | **0.05** |
| ***Liver function*** |  |  |
| ALT (U/L) | 0.025 (0.014, 0.037) | **1.70e-4** |
| AST (U/L) | 0.005 (-0.007, 0.017) | 0.56 |
| Alkaline Phosphatase (U/L) | 0.029 (0.016, 0.042) | **8.74e-5** |
| Gamma-GT (U/L) | 0.039 (0.027, 0.051) | **1.64e-9** |
| ***Kidney function*** |  |  |
| Creatinine (µmol/L) | -0.011 (-0.018, -0.003) | **0.03** |
| Creatinine 24hrs urine (mmol/L) | 0.018 (0.010, 0.026) | **1.10e-4** |
| Estimated Glomerular Filtration Rate (eGFR) | 0.008 (0.001, 0.016) | 0.09 |
| Urinary albumin excretion (UAE) 24 hrs | -0.009 (-0.021, 0.004) | 0.34 |
| Urinary albumin-creatinine ratio (UACR) | 0.001 (-0.011, 0.014) | 0.94 |
| Uric acid (mmol/L) | 0.022 (0.012, 0.032) | **3.72e-4** |
| ***Thyroid*** |  |  |
| Free T3 (pmol/L) | 0.002 (-0.013, 0.018) | 0.87 |
| Free T4 (pmol/L) | 0.004 (-0.013, 0.022) | 0.78 |
| TSH (mU/L) | -0.021 (-0.039, -0.003) | 0.07 |
| ***Inflammation*** |  |  |
| hsCRP (mg/L) | 0.028 (0.014, 0.041) | **3.76e-4** |
| Leukocytes (10^9/L) | 0.040 (0.030, 0.049) | **1.24e-14** |
| ***Blood pressure*** |  |  |
| Systolic BP | 0.016 (0.007, 0.025) | **1.84e-3** |
| Diastolic BP | 0.018 (0.010, 0.027) | **3.77e-4** |

Note: Adults with self-reported ADHD were excluded from these analyses (N=383). P_adj_ = FDR-adjusted p values, statistically significant (p<0.05) in bold.

# Supplementary Table 7. Associations between cardiometabolic outcomes and ADHD-PGS: additionally adjusting for BMI

| **Condition** | **OR (95% CI)** | **P_adj_ value** |
| --- | --- | --- |
| ***Metabolic disease*** | 1.021 (1.00, 1.045) | 0.23 |
| Type 2 diabetes | 1.168 (1.103, 1.236) | **1.84e-6** |
| Hypertension | 1.003 (0.98, 1.03) | 0.89 |
| ***Cardiovascular disease*** | 1.082 (1.038, 1.130) | **1.70e-3** |
| Thrombosis | 1.136 (1.045, 1.235) | **0.01** |
| History of coronary artery bypass grafting (CABG) | 1.118 (1.033, 1.211) | **0.03** |
| MI | 1.080 (0.983, 1.188) | 0.25 |
| Atherosclerosis | 1.130 (1.00, 1.281) | 0.15 |
| Stroke | 1.06 (0.948, 1.181) | 0.50 |
| Narrowing carotid arteries | 1.069 (0.905, 1.262) | 0.62 |
| Heart failure | 1.007 (0.881, 1.151) | 0.95 |
| Atrial fibrillation | 0.993 (0.873, 1.130) | 0.95 |
| Heart valve disorders | 1.015 (0.925, 1.116) | 0.87 |
| Aneurysm | 0.973 (0.817, 1.159) | 0.87 |

Note: P_adj_ = FDR-adjusted p values, statistically significant in bold. Obesity not included in Metabolic disease definition.

# Supplementary Table 8. Associations between cardiometabolic biomarkers and ADHD-PGS: additionally adjusting for BMI

| **Measure** | **Standardized beta (95% CI)** | **P_adj_ value** |
| --- | --- | --- |
| ***Glucose metabolic*** |  |  |
| Glucose (mmol/L) | 0.007 (-0.001, 0.016) | 0.21 |
| HbA1c (%) | 0.009 (0.001, 0.018) | 0.11 |
| ***Red blood cells*** |  |  |
| Hematocrit (v/v) | 0.005 (-0.002, 0.012) | 0.33 |
| Hemoglobin (mmol/L) | 0.005 (-0.002, 0.012) | 0.32 |
| ***Lipids*** |  |  |
| Total cholesterol (mmol/L) | 0.008 (-0.001, 0.017) | 0.13 |
| LDL cholesterol (mmol/L) | 0.007 (-0.001, 0.016) | 0.22 |
| HDL cholesterol (mmol/L) | -0.017 (-0.025, -0.009) | **4.79e-4** |
| Triglycerides (mmol/L) | 0.017 (0.008, 0.026) | **1.20e-3** |
| Apolipo A1 in serum (g/L) | -0.008 (-0.033, 0.017) | 0.73 |
| Apolipo B100 (g/L) | 0.020 (-0.005, 0.046) | 0.25 |
| ***Liver function*** |  |  |
| ALT (U/L) | 0.009 (-0.002, 0.020) | 0.22 |
| AST (U/L) | 0.002 (-0.010, 0.014) | 0.88 |
| Alkaline Phosphatase (U/L) | 0.015 (0.002, 0.027) | 0.07 |
| Gamma-GT (U/L) | 0.023 (0.011, 0.034) | **7.10e-4** |
| ***Kidney function*** |  |  |
| Creatinine (µmol/L) | -0.010 (-0.018, -0.002) | **0.04** |
| Creatinine 24hrs urine (mmol/L) | 0.006 (-0.002, 0.014) | 0.26 |
| Estimated Glomerular Filtration Rate (eGFR) | 0.009 (0.001, 0.016) | 0.07 |
| Urinary albumin excretion (UAE) 24 hrs | -0.011 (-0.024, 0.002) | 0.21 |
| Urinary albumin-creatinine ratio (UACR) | -0.001 (-0.014, 0.012) | 0.95 |
| Uric acid (mmol/L) | 0.001 (-0.009, 0.011) | 0.94 |
| ***Thyroid*** |  |  |
| Free T3 (pmol/L) | 0.001 (-0.015, 0.017) | 0.96 |
| Free T4 (pmol/L) | 0.001 (-0.004, 0.030) | 0.27 |
| TSH (mU/L) | -0.022 (-0.040, -0.004) | **0.04** |
| ***Inflammation*** |  |  |
| hsCRP (mg/L) | 0.002 (-0.010, 0.015) | 0.85 |
| Leukocytes (10^9/L) | 0.025 (0.016, 0.035) | **1.15e-6** |
| ***Blood pressure*** |  |  |
| Systolic BP | -0.002 (-0.010, 0.005) | 0.73 |
| Diastolic BP | -0.002 (-0.010, 0.005) | 0.73 |

Note: P_adj_ = FDR-adjusted p values, statistically significant (p<0.05) in bold.

# Supplementary Table 9. Associations between cardiometabolic diseases and ADHD-PGS: stratified by sex

|  | **FEMALES (N=29 675)** | | **MALES (N=21 093)** | |  |
| --- | --- | --- | --- | --- | --- |
| **Condition** | **Prevalence (%)** | **OR (95% CI)** | **Prevalence (%)** | **OR (95% CI)** | **P_adj_ PGS*sex interaction** |
| ***ADHD*** | 184 (0.67%) | 1.332 (1.151, 1.542) | 199 (1.03%) | 1.389 (1.209, 1.596) | 0.95 |
| ***Metabolic disease*** | 8 768 (31.74%) | 1.086 (1.057, 1.116) | 5 465 (28.42%) | 1.133 (1.095, 1.172) | 0.07 |
| Type 2 diabetes | 654 (2.21%) | 1.178 (1.085, 1.279) | 691 (3.29%) | 1.295 (1.199, 1.399) | 0.21 |
| Obesity | 2470 (11.75%) | 1.147 (1.109, 1.187) | 4 215 (14.25%) | 1.203 (1.153, 1.256) | 0.15 |
| Hypertension | 6 028 (21.82%) | 1.027 (0.996, 1.059) | 3 593 (18.68%) | 1.058 (1.017, 1.099) | 0.23 |
| ***Cardiovascular disease*** | 1 151 (4.14%) | 1.152 (1.084, 1.224) | 1 299 (6.71%) | 1.062 (0.999, 1.129) | 0.12 |
| Thrombosis | 427 (1.53%) | 1.262 (1.146, 1.389) | 171 (0.88%) | 0.966 (0.830, 1.123) | **0.02** |
| History of coronary artery bypass grafting (CABG) | 161 (0.58%) | 1.229 (1.050, 1.440) | 497 (2.57%) | 1.121 (1.021, 1.231) | 0.42 |
| MI | 107 (0.38%) | 1.118 (1.002, 1.247) | 342 (1.77%) | 1.092 (0.900, 1.324) | 0.95 |
| Atherosclerosis | 88 (0.32%) | 1.162 (0.947, 1.425) | 138 (0.71%) | 1.136 (0.969, 1.331) | 0.88 |
| Stroke | 159 (0.57%) | 1.022 (0.874, 1.195) | 185 (0.95%) | 1.122 (0.962, 1.310) | 0.62 |
| Narrowing carotid arteries | 51 (0.18%) | 1.168 (0.891, 1.531) | 64 (0.33%) | 1.026 (0.832, 1.264) | 0.55 |
| Heart failure | 95 (0.34%) | 1.131 (9.092, 1.407) | 147 (0.76%) | 0.963 (0.812, 1.142) | 0.42 |
| Atrial fibrillation | 83 (0.30%) | 1.181 (0.941, 1.483) | 159 (0.82%) | 0.942 (0.808, 1.100) | 0.27 |
| Heart valve disorders | 261 (0.94%) | 1.085 (0.954, 1.234) | 231 (1.19%) | 0.933 (0.812, 1.072) | 0.26 |
| Aneurysm | 29 (0.10%) | 1.067 (0.729, 1.558) | 101 (0.52%) | 0.968 (0.794, 1.181) | 0.83 |

Note: Analyses adjusting for genotyping chip, 8 PCs, PC*chip, age, and cluster by fam_ID (children, parents, siblings, partners). P_adj_ = FDR-adjusted p values, statistically significant (p<0.05) in bold.

# Supplementary Table 10. Associations between cardiometabolic biological risk indicators and ADHD-PGS: stratified by sex

|  | **FEMALES (N=27 818)** | | **MALES (N=19 371)** | |  |
| --- | --- | --- | --- | --- | --- |
| **Measure** | **Mean (SD)** | **Standardized beta**  **(95% CI)** | **Mean (SD)** | **Standardized beta**  **(95% CI)** | **P_adj_ PGS*sex interaction** |
| ***Glucose metabolic*** |  |  |  |  |  |
| Glucose (mmol/L) | 4.80 (0.51) | 0.020 (0.009, 0.032) | 5.06 (0.53) | 0.034 (0.020, 0.048) | 0.26 |
| HbA1c (%) | 5.48 (0.32) | 0.016 (0.005, 0.027) | 5.50 (0.32) | 0.021 (0.007, 0.035) | 0.74 |
| ***Red blood cells*** |  |  |  |  |  |
| Hematocrit (v/v) | 0.40 (0.03) | 0.013 (0.004, 0.023) | 0.45 (0.02) | 0.006 (-0.005, 0.017) | 0.50 |
| Hemoglobin (mmol/L) | 8.33 (0.57) | 0.012 (0.003, 0.020) | 9.38 (0.58) | 0.007 (-0.004, 0.017) | 0.63 |
| ***Lipid metabolism*** |  |  |  |  |  |
| Total cholesterol (mmol/L) | 5.05 (1.02) | 0.015 (0.004, 0.025) | 5.15 (1.01) | 0.009 (-0.005, 0.022) | 0.65 |
| LDL cholesterol (mmol/L) | 3.14 (0.93) | 0.019 (0.008, 0.030) | 3.42 (0.92) | 0.009 (-0.004, 0.023) | 0.42 |
| HDL cholesterol (mmol/L) | 1.60 (0.38) | -0.035 (-0.046, -0.023) | 1.31 (0.31) | -0.050 (-0.063, -0.038) | 0.15 |
| Triglycerides (mmol/L) | 1.02 (0.52) | 0.036 (0.025, 0.047) | 1.30 (0.69) | 0.040 (0.024, 0.055) | 0.86 |
| Apolipo A1 in serum (g/L) | 1.69 (0.28) | -0.042 (-0.079, -0.006) | 1.51 (0.23) | 0.004 (-0.030, 0.038) | 0.28 |
| Apolipo B100 (g/L) | 0.88 (0.22) | 0.033 (-0.001, 0.067) | 1.00 (0.24) | 0.041 (0.001, 0.081) | 0.89 |
| ***Liver function*** |  |  |  |  |  |
| ALT (U/L) | 18.32 (9.16) | 0.013 (-0.002, 0.027) | 28.80 (13.48) | 0.036 (0.018, 0.054) | 0.09 |
| AST (U/L) | 22.03 (5.72) | -0.005 (-0.020, 0.010) | 26.82 (6.87) | 0.014 (-0.006, 0.033) | 0.23 |
| Alkaline Phosphatase (U/L) | 60.15 (17.28) | 0.030 (0.014, 0.047) | 65.27 (16.11) | 0.021 (0.002, 0.039) | 0.63 |
| Gamma-GT (U/L) | 20.35 (12.80) | 0.030 (0.015, 0.045) | 31.54 (18.13) | 0.049 (0.030, 0.068) | 0.27 |
| ***Kidney function*** |  |  |  |  |  |
| Creatinine (µmol/L) | 67.20 (9.29) | -0.012 (-0.022, -0.002) | 82.39 (10.68) | -0.009 (-0.020, 0.003) | 0.55 |
| Creatinine 24hrs urine (mmol/L) | 6.85 (3.28) | 0.015 (0.005, 0.026) | 10.13 (3.96) | 0.025 (0.013, 0.037) | 0.42 |
| Estimated Glomerular Filtration Rate (eGFR) | 98.88 (15.09) | 0.010 (-0.0002, 0.020) | 101.41 (14.91) | 0.006 (-0.004, 0.016) | 0.53 |
| Urinary albumin excretion (UAE) 24 hrs | 7.37 (16.64) | -0.014 (-0.023, 0.001) | 8.52 (23.18) | 0.001 (-0.020, 0.023) | 0.37 |
| Urinary albumin-creatinine ratio (UACR) | 3.44 (8.13) | -0.006 (-0.023, 0.010) | 3.39 (9.55) | 0.013 (-0.007, 0.034) | 0.28 |
| Uric acid (mmol/L) | 0.26 (0.06) | 0.025 (0.012, 0.038) | 0.34 (0.06) | 0.015 (-0.002, 0.031) | 0.75 |
| ***Thyroid*** |  |  |  |  |  |
| Free T3 (pmol/L) | 5.06 (0.62) | -0.011 (-0.032, 0.010) | 5.47 (0.60) | 0.023 (-0.097, 0.046) | 0.10 |
| Free T4 (pmol/L) | 15.50 (2.08) | 0.0002 (-0.022, 0.022) | 16.16 (2.11) | 0.009 (-0.018, 0.036) | 0.87 |
| TSH (mU/L) | 2.58 (1.72) | -0.024 (-0.047, 0.0001) | 2.31 (1.35) | -0.020 (-0.045, 0.005) | 0.87 |
| ***Inflammation*** |  |  |  |  |  |
| hsCRP (mg/L) | 2.55 (3.09) | 0.023 (0.005, 0.041) | 1.89 (2.55) | 0.037 (0.018, 0.057) | 0.56 |
| Leukocytes (10^9/L) | 6.05 (1.61) | 0.046 (0.033, 0.058) | 5.97 (1.53) | 0.036 (0.022, 0.051) | 0.36 |
| ***Blood pressure*** |  |  |  |  |  |
| Systolic BP | 123.07 (16.58) | 0.013 (0.002, 0.024) | 131.64 (15.18) | 0.017 (0.005, 0.030) | 0.73 |
| Diastolic BP | 72.33 (9.55) | 0.015 (0.004, 0.026) | 77.05 (10.15) | 0.025 (0.011, 0.038) | 0.38 |

Note: Analyses adjusting for genotyping chip, 8 PCs, PC*chip, sex, age, and cluster by fam_ID (children, parents, siblings, partners). Values > 4 SDs are excluded. P_adj_ = FDR-adjusted p values, statistically significant (p<0.05) in bold.

#

# Supplementary Table 11. Associations between cardiometabolic diseases and ADHD-PGS: stratified by age group

|  | **YOUNGER COHORT (<=45 years) (N=25 144)** | | **OLDER COHORT (>45 years) (N=22 047)** | |  |
| --- | --- | --- | --- | --- | --- |
| **Condition** | **Prevalence (%)** | **OR (95% CI)** | **Prevalence (%)** | **OR (95% CI)** | **P_adj_ PGS*age interaction** |
| ***ADHD*** | 293 (1.18%) | 1.425 (1.273, 1.596) | 99 (0.05%) | 1.152 (0.937, 1.415) | 0.21 |
| ***Metabolic disease*** | 5259 (21.13%) | 1.127 (1.092, 1.163) | 8925 (40.75%) | 1.085 (1.055, 1.116) | 0.06 |
| Type 2 diabetes | 189 (0.75%) | 1.115 (0.973, 1.278) | 1140 (5.18%) | 1.258 (1.183, 1.337) | 0.36 |
| Obesity | 2981 (11.86%) | 1.175 (1.130, 1.222) | 3678 (16.69%) | 1.159 (1.117, 1.203) | 0.67 |
| Hypertension | 2984 (11.97%) | 1.072 (1.031, 1.115) | 6628 (30.25%) | 1.019 (0.988, 1.050) | 0.06 |
| ***Cardiovascular disease*** | 461 (1.83%) | 1.134 (1.031, 1.247) | **1989 (9.02%)** | 1.097 (1.046, 1.151) | 0.51 |
| Thrombosis | 154 (0.61%) | 1.287 (1.097, 1.510) | **444 (2.01%)** | 1.128 (1.026, 1.242) | 0.21 |
| History of coronary artery bypass grafting (CABG) | 41 (0.16%) | 1.220 (0.909, 1.639) | 617 (2.80%) | 1.136 (1.047, 1.233) | 0.63 |
| MI | 30 (0.12%) | 1.191 (0.827, 1.715) | 419 (1.90%) | 1.099 (0.998, 1.212) | 0.78 |
| Atherosclerosis | 23 (0.09%) | 1.137 (0.679, 1.905) | 203 (0.92%) | 1.141 (1.005, 1.294) | 0.99 |
| Stroke | 69 (0.27%) | 1.053 (0.822, 1.348) | 275 (1.25%) | 1.084 (0.959, 1.226) | 0.91 |
| Narrowing carotid arteries | 19 (0.08%) | 0.832 (0.549, 1.259) | 96 (0.44%) | 1.156 (0.965, 1.385) | 0.27 |
| Heart failure | 23 (0.09%) | 1.204 (0.821, 1.767) | 219 (0.99%) | 1.009 (0.875, 1.163) | 0.57 |
| Atrial fibrillation | 10 (0.04%) | 1.184 (0.797, 1.759) | 232 (1.05%) | 1.013 (0.887, 1.157) | 0.67 |
| Heart valve disorders | 162 (0.64%) | 1.033 (0.875, 1.220) | 330 (1.50%) | 1.003 (0.895, 1.124) | 0.89 |
| Aneurysm | 12 (0.05%) | 0.925 (0.552, 1.551) | 118 (0.54%) | 0.993 (0.824, 1.196) | 0.90 |

Note: Analyses adjusting for genotyping chip, 8 PCs, PC*chip, sex, age, and cluster by fam_ID (children, parents, siblings, partners). P_adj_ = FDR-adjusted p values, statistically significant (p<0.05) in bold.

# Supplementary Table 12. Associations between cardiometabolic biological risk indicators and ADHD-PGS: stratified by age group

|  | **YOUNGER COHORT (<=45 years) (N=25 144)** | | **OLDER COHORT (>45 years) (N=22 047** | | |
| --- | --- | --- | --- | --- | --- |
| **Measure** | **Mean (SD)** | **Standardized beta**  **(95% CI)** | **Mean (SD)** | **Standardized beta (95% CI)** | **P_adj_ PGS*age interaction** |
| ***Glucose metabolic*** |  |  |  |  |  |
| Glucose (mmol/L) | 4.78 (0.46) | 0.011 (0.001, 0.023) | 5.06 (0.57) | 0.040 (0.026, 0.055) | **0.04** |
| HbA1c (%) | 5.38 (0.29) | 0.014 (0.002, 0.026) | 5.62 (0.32) | 0.026 (0.013, 0.039) | 0.42 |
| ***Red blood cells*** |  |  |  |  |  |
| Hematocrit (v/v) | 0.42 (0.03) | 0.010 (0.001, 0.020) | 0.42 (0.03) | 0.011 (0.001, 0.022) | 0.86 |
| Hemoglobin (mmol/L) | 8.73 (0.79) | 0.009 (-0.001, 0.019) | 8.79 (0.74) | 0.009 (-0.001, 0.019) | 0.95 |
| ***Lipid metabolism*** |  |  |  |  |  |
| Total cholesterol (mmol/L) | 4.69 (0.89) | 0.013 (0.002, 0.024) | 5.52 (0.98) | 0.008 (-0.005, 0.021) | 0.50 |
| LDL cholesterol (mmol/L) | 2.93 (0.83) | 0.018 (0.007, 0.029) | 3.60 (0.91) | 0.009 (-0.004, 0.022) | 0.28 |
| HDL cholesterol (mmol/L) | 1.44 (0.35) | -0.037 (-0.049, -0.025) | 1.53 (0.41) | -0.047 (-0.060, -0.034) | 0.46 |
| Triglycerides (mmol/L) | 1.04 (0.57) | 0.025 (0.013, 0.038) | 1.23 (0.63) | 0.050 (0.037, 0.063) | **0.04** |
| Apolipo A1 in serum (g/L) | 1.60 (0.27) | -0.018 (-0.050, 0.014) | 1.65 (0.28) | -0.040 (-0.086, 0.005) | 0.86 |
| Apolipo B100 (g/L) | 0.90 (0.24) | 0.039 (0.006, 0.072) | 0.99 (0.23) | 0.039 (-0.006, 0.084) | 0.97 |
| ***Liver function*** |  |  |  |  |  |
| ALT (U/L) | 21.79 (12.75) | 0.019 (0.003, 0.036) | 23.38 (11.66) | 0.025 (0.009, 0.041) | 0.64 |
| AST (U/L) | 23.20 (6.62) | -0.001 (-0.018, 0.017) | 24.80 (6.60) | 0.009 (-0.008, 0.026) | 0.38 |
| Alkaline Phosphatase (U/L) | 59.63 (16.41) | 0.030 (0.012, 0.048) | 64.94 (17.18) | 0.023 (0.006, 0.041) | 0.82 |
| Gamma-GT (U/L) | 22.93 (14.89) | 0.026 (0.001, 0.043) | 26.85 (17.14) | 0.047 (0.030, 0.064) | 0.26 |
| ***Kidney function*** |  |  |  |  |  |
| Creatinine (µmol/L) | 72.52 (11.54) | -0.016 (-0.026, -0.006) | 74.41 (13.20) | -0.005 (-0.017, 0.006) | 0.34 |
| Creatinine 24hrs urine (mmol/L) | 9.06 (4.10) | 0.026 (0.015, 0.038) | 7.27 (3.49) | 0.012 (0.002, 0.023) | 0.33 |
| eGFR | 107.50 (13.17) | 0.014 (0.004. 0.024) | 91.81 (12.77) | 0.003 (-0.007, 0.014) | 0.97 |
| Urinary albumin excretion (UAE) 24 hrs | 7.09 (15.68) | -0.004 (-0.021, 0.014) | 8.56 (22.60) | -0.011 (-0.030, 0.007) | 0.58 |
| Urinary albumin-creatinine ratio (UACR) | 2.81 (6.14) | 0.013 (-0.003, 0.030) | 4.00 (10.57) | -0.010 (-0.030, 0.010) | 0.13 |
| Uric acid (mmol/L) | 0.29 (0.07) | 0.011 (-0.002, 0.025) | 0.30 (0.07) | 0.032 (0.017, 0.047) | 0.11 |
| ***Thyroid*** |  |  |  |  |  |
| Free T3 (pmol/L) | 5.37 (0.65) | 0.007 (-0.015, 0.029) | 5.10 (0.61) | 0.001 (-0.022, 0.023) | 0.87 |
| Free T4 (pmol/L) | 15.87 (2.10) | 0.014 (-0.009, 0.038) | 15.68 (2.13) | -0.001 (-0.026, 0.023) | 0.87 |
| TSH (mU/L) | 2.39 (1.49) | -0.015 (-0.039, 0.009) | 2.55 (1.67) | -0.029 (-0.054, -0.003) | 0.58 |
| ***Inflammation*** |  |  |  |  |  |
| hsCRP (mg/L) | 2.30 (3.01) | 0.026 (0.006, 0.046) | 2.26 (2.80) | 0.033 (0.015, 0.051) | 0.88 |
| Leukocytes (10^9/L) | 6.10 (1.57) | 0.040 (0.027, 0.053) | 5.92 (1.57) | 0.044 (0.030, 0.057) | 0.88 |
| ***Blood pressure*** |  |  |  |  |  |
| Systolic BP | 121.51 (13.43) | 0.013 (0.004, 0.023) | 132.10 (17.85) | 0.017 (0.003, 0.031) | 0.97 |
| Diastolic BP | 71.27 (8.74) | 0.014 (0.003, 0.025) | 7.42 (10.45) | 0.021 (0.007, 0.035) | 0.99 |

Note: Analyses adjusting for genotyping chip, 8 PCs, PC*chip, sex, age, and cluster by fam_ID (children, parents, siblings, partners). Values > 4 SDs are excluded. P_adj_ = FDR-adjusted p values, statistically significant (p<0.05) in bold.

#

# Supplementary Table 13. Associations between cardiometabolic diseases and ADHD-PGS: stratified by educational attainment

|  | **LOW EDUCATIONAL ATTAINMENT (N=31 493)** | | **HIGH EDUCATIONAL ATTAINMENT (N=13 460)** | |  |
| --- | --- | --- | --- | --- | --- |
| **Condition** | **Prevalence (%)** | **OR (95% CI)** | **Prevalence (%)** | **OR (95% CI)** | **P_adj_ PGS*SES interaction** |
| ***ADHD*** | 290 (0.92%) | 1.376 (1.231, 1.538) | 79 (0.59%) | 1.234 (0.954, 1.595) | 0.60 |
| ***Metabolic disease*** | 10364 (32.96%) | 1.090 (1.063, 1.119) | 3173 (23.64%) | 1.088 (1.043, 1.135) | 0.74 |
| Type 2 diabetes | 997 (3.17%) | 1.210 (1.135, 1.291) | 201 (1.50%) | 1.276 (1.113, 1.464) | 0.73 |
| Obesity | 5040 (16.01%) | 1.140 (1.106, 1.177) | 1223 (9.09%) | 1.192 (1.123, 1.266) | 0.55 |
| Hypertension | 7032 (22.33%) | 1.040 (1.010, 1.070) | 2298 (17.07%) | 1.018 (0.970, 1.067) | 0.46 |
| ***Cardiovascular disease*** | 1723 (5.47%) | 1.093 (1.038, 1.150) | 511 (3.80%) | 1.172 (1.067, 1.287) | 0.37 |
| Thrombosis | 447 (1.42%) | 1.147 (1.044, 1.261) | 118 (0.88%) | 1.309 (1.083, 1.581) | 0.34 |
| History of coronary artery bypass grafting (CABG) | 476 (1.51%) | 1.122 (1.021, 1.232) | 119 (0.88%) | 1.213 (1.016, 1.450) | 0.74 |
| MI | 322 (1.02%) | 1.038 (0.929, 1.160) | 78 (0.58%) | 1.348 (1.055, 1.722) | 0.13 |
| Atherosclerosis | 166 (0.53%) | 1.196 (1.040, 1.375) | 44 (0.33%) | 1.010 (0.767, 1.331) | 0.42 |
| Stroke | 244 (0.78%) | 1.055 (0.931, 1.195) | 63 (0.47%) | 1.180 (0.875, 1.590) | 0.80 |
| Narrowing carotid arteries | 88 (0.28%) | 1.164 (0.969, 1.398) | 23 (0.17%) | 0.827 (0.594, 1.152) | 0.13 |
| Heart failure | 172 (0.55%) | 1.036 (0.890, 1.205) | 43 (0.32%) | 1.032 (0.721, 1.478) | 0.95 |
| Atrial fibrillation | 156 (0.50%) | 1.000 (0.850, 1.176) | 57 (0.42%) | 1.065 (0.818, 1.385) | 0.74 |
| Heart valve disorders | 339 (1.08%) | 1.074 (0.958, 1.204) | 125 (0.93%) | 0.912 (0.761, 1.094) | 0.40 |
| Aneurysm | 93 (0.30%) | 0.998 (0.820, 1.215) | 24 (0.18%) | 1.045 (0.722, 1.514) | 0.91 |

Low educational attainment level includes: no education, primary education, secondary vocational education, general secondary education. High educational attainment level includes: higher vocational education and university education. P_adj_ = FDR-adjusted p values, statistically significant (p<0.05) in bold.

# Supplementary Table 14. Associations between cardiometabolic biological risk indicators and ADHD-PGS: stratified by educational attainment

|  | **LOW EDUCATIONAL ATTAINMENT (N=31 472)** | | **HIGH EDUCATIONAL ATTAINMENT (N=13 454)** | |  |
| --- | --- | --- | --- | --- | --- |
| **Measure** | **Mean (SD)** | **Standardized beta**  **(95% CI)** | **Mean (SD)** | **Standardized beta (95% CI)** | **P_adj_ PGS*SES interaction** |
| ***Glucose metabolic*** |  |  |  |  |  |
| Glucose (mmol/L) | 4.93 (0.53) | 0.020 (0.010, 0.031) | 4.84 (0.49) | 0.028 (0.013, 0.044) | 0.58 |
| HbA1c (%) | 5.50 (0.32) | 0.019 (0.008, 0.029) | 5.44 (0.31) | 0.012 (-0.004, 0.028) | 0.63 |
| ***Red blood cells*** |  |  |  |  |  |
| Hematocrit (v/v) | 0.42 (0.03) | 0.010 (0.002, 0.019) | 0.42 (0.03) | 0.002 (-0.011, 0.016) | 0.42 |
| Hemoglobin (mmol/L) | 8.77 (0.77) | 0.009 (0.001, 0.017) | 8.75 (0.78) | 0.005 (-0.008, 0.018) | 0.72 |
| ***Lipids*** |  |  |  |  |  |
| Total cholesterol (mmol/L) | 5.13 (1.03) | 0.013 (0.002. 0.023) | 4.98 (0.98) | 0.011 (-0.004, 0.026) | 0.88 |
| LDL cholesterol (mmol/L) | 3.29 (0.94) | 0.016 (0.005, 0.027) | 3.15 (0.90) | 0.014 (-0.001, 0.029) | 0.85 |
| HDL cholesterol (mmol/L) | 1.47 (0.38) | -0.038 (-0.049, -0.028) | 1.51 (0.38) | -0.034 (-0.049, -0.018) | 0.56 |
| Triglycerides (mmol/L) | 1.16 (0.62) | 0.036 (0.024, 0.047) | 1.06 (0.57) | 0.027 (0.011, 0.043) | 0.44 |
| Apolipo A1 in serum (g/L) | 1.61 (0.28) | -0.019 (-0.049, 0.010) | 1.63 (0.28) | -0.038 (-0.089, 0.013) | 0.82 |
| Apolipo B100 (g/L) | 0.94 (0.24) | 0.028 (-0.004, 0.060) | 0.90 (0.23) | 0.037 (-0.011, 0.083) | 0.99 |
| ***Liver function*** |  |  |  |  |  |
| ALT (U/L) | 22.65 (12.29) | 0.031 (0.017, 0.045) | 22.54 (12.33) | 0.006 (-0.016, 0.027) | 0.12 |
| AST (U/L) | 23.93 (6.68) | 0.010 (-0.005, 0.025) | 24.09 (6.56) | -0.007 (-0.029, 0.015) | 0.42 |
| Alkaline Phosphatase (U/L) | 63.08 (17.23) | 0.025 (0.009, 0.040) | 59.77 (16.13) | 0.016 (-0.007, 0.040) | 0.50 |
| Gamma-GT (U/L) | 25.29 (16.48) | 0.039 (0.025, 0.053) | 23.90 (15.29) | 0.027 (0.006, 0.050) | 0.41 |
| ***Kidney function*** |  |  |  |  |  |
| Creatinine (µmol/L) | 72.99 (12.41) | -0.014 (-0.024, -0.005) | 74.42 (12.12) | 0.003 (-0.011, 0.016) | 0.13 |
| Creatinine 24hrs urine (mmol/L) | 8.29 (3.97) | 0.016 (0.006, 0.026) | 8.14 (3.83) | 0.008 (-0.006, 0.023) | 0.44 |
| eGFR | 99.90 (15.33) | 0.011 (0.003, 0.020) | 100.80 (14.02) | -0.004 (-0.018, 0.009) | 0.15 |
| Urinary albumin excretion (UAE) 24 hrs | 7.92 (20.09) | -0.016 (-0.032, -0.001) | 7.30 (17.22) | 0.006 (-0.017, 0.029) | 0.21 |
| Urinary albumin-creatinine ratio (UACR) | 3.52 (8.98) | -0.009 (-0.025, 0.007) | 2.88 (6.85) | 0.021 (-0.001, 0.042) | 0.07 |
| Uric acid (mmol/L) | 0.29 (0.07) | 0.021 (0.008, 0.034) | 0.29 (0.07) | 0.012 (-0.007, 0.030) | 0.63 |
| ***Thyroid*** |  |  |  |  |  |
| Free T3 (pmol/L) | 5.24 (0.65) | 0.001 (-0.019, 0.021) | 5.21 (0.62) | -0.016 (-0.044, 0.012) | 0.46 |
| Free T4 (pmol/L) | 15.71 (2.12) | 0.008 (-0.013, 0.029) | 15.87 (2.08) | 0.003 (-0.028, 0.033) | 0.85 |
| TSH (mU/L) | 2.47 (1.62) | -0.018 (-0.040, 0.003) | 2.45 (1.49) | -0.012 (-0.044, 0.020) | 0.94 |
| ***Inflammation*** |  |  |  |  |  |
| hsCRP (mg/L) | 2.40 (3.01) | 0.033 (0.017, 0.049) | 1.96 (2.59) | -0.011 (-0.037, 0.014) | **0.04** |
| Leukocytes (10^9/L) | 6.10 (1.62) | 0.040 (0.028, 0.052) | 5.83 (1.46) | 0.022 (0.006, 0.039) | 0.12 |
| ***Blood pressure*** |  |  |  |  |  |
| Systolic BP | 127.55 (16.74) | 0.009 (-0.002, 0.019) | 123.77 (15.47) | 0.012 (-0.002, 0.027) | 0.96 |
| Diastolic BP | 74.64 (10.22) | 0.017 (0.006, 0.028) | 73.30 (9.70) | 0.013 (-0.002, 0.028) | 0.63 |

Low educational attainment level includes: no education, primary education, secondary vocational education, general secondary education. High educational attainment level includes: higher vocational education and university education. P_adj_ = FDR-adjusted p values, statistically significant (p<0.05) in bold.
